# Perception of prescribing factors and purchase statistics of non-steroidal anti-inflammatory drugs in an orthopedic clinic

**DOI:** 10.1186/s13104-020-04949-y

**Published:** 2020-02-24

**Authors:** Jan Schjøtt, Hilde Erdal, Sofie Opskar, Tormod K. Bjånes

**Affiliations:** 1 Regional Medicines Information and Pharmacovigilance Centre (RELIS Vest), Haukeland University Hospital, Bergen, Norway; 2 Department of Medical Biochemistry and Pharmacology, Haukeland University Hospital, Bergen, Norway; 3 Department of Clinical Science, Faculty of Medicine and Dentistry, University of Bergen, Bergen, Norway

**Keywords:** Non-steroidal anti-inflammatory drugs, Hospital, Orthopedic

## Abstract

**Objectives:**

Nonsteroidal anti-inflammatory drugs (NSAIDS) are associated with concern of adverse drug reactions (ADRS) including gastrointestinal, cardiovascular, renal, and musculoskeletal. Non-selective and selective NSAIDS are proposed to differ with regard to their potential to cause ADRS. The aim of this pilot study was to compare perception of prescribing factors and purchase statistics of NSAIDS among physicians in a Norwegian orthopedic clinic.

**Results:**

Forty-five (55%) of 82 invited physicians from the orthopedic clinic participated anonymous in a survey in February 2017. Effect and ADRS were rated as the most important factors for prescribing of NSAIDS. The participants were equally concerned about specific ADRS for prescription of non-selective and selective NSAIDS irrespective of type of ADR. They were generally more concerned about cardiovascular, gastrointestinal and renal ADRS than musculoskeletal. Purchase statistics from 2015 and 2016 showed that celecoxib, a selective NSAID, dominated in the orthopedic clinic. The discrepancy between perception of prescribing factors and purchase statistics of NSAIDS was possibly explained by a high degree of conformity to clinic guidelines. Our preliminary results indicate that perception of prescribing factors of NSAIDS among orthopedics should be surveyed in multicenter or multinational studies.

## Introduction

Nonsteroidal anti-inflammatory drugs (NSAIDS) are among of the most commonly used drug classes in the world [1]. In Norway about 800,000 individuals received prescriptions of NSAIDS annually over the last 10 years [2]. NSAIDS are important in multimodal postoperative pain management in hospitals, including orthopedic departments [3]. NSAIDS show different relative affinities for cyclooxygenase-1 (COX-1) and cyclooxygenase-2 (COX-2) isoenzymes which might explain adverse drug reaction (ADR) profiles of the drugs [4, 5]. Non-selective NSAIDS have been associated with gastrointestinal ADRS while selective (COX-2 inhibitors) like coxibs and diclofenac have been associated with cardiovascular ADRS [4, 5]. NSAIDS are also associated with renal and musculoskeletal ADRS. In spite of numerous experimental and animal data on impaired healing of fractures or soft tissue, there is no definitive evidence in humans [6–8]. There is also incomplete clinical evidence with regard to risk of cardiovascular disease with use of NSAIDS, non-selective or selective [9]. In hospitals, orthopedic physicians are frequent prescribers of NSAIDS, but little is known about their perception of prescribing factors including ADRS. This subject is of clinical relevance due to the significant number of hospitalizations and deaths attributed to NSAIDS worldwide.

## Main text

### Methods

#### Study population

All physicians (n = 82) working in the Orthopedic Clinic, Haukeland University Hospital, Bergen, Norway were invited by e-mail to participate in a survey during February 2017. Participant anonymity was ensured throughout the survey, and automatic e-mail reminders were sent to non-responders 13 and 24 days after the initial invitation without unmasking their identities. To stimulate enrollment, scratch lottery tickets were drawn among participants.

#### Survey

E-mail address to all the physicians in the orthopedic clinic was collected for recruitment, imported into an in-house electronic survey program, and anonymized. The researchers were also blinded for the responders and non-responders. The physicians were asked to rate different factors of importance for prescribing of NSAIDS. The factors included effect, ADRS, risk factors like patient age, drug interactions, comorbidity, written guidelines, routines and treatment traditions, and advertising from the pharmaceutical industry in the orthopedic clinic. The physicians also rated non-selective and selective NSAIDs with regard to risk perception of ADRS in different organs or tissues. Rating included categories as *not at all, to a small degree, to some degree, to a large degree and to a very large degree*. The physicians were asked about working experience (e.g. < 2 years; 2–4 years; 5–10 years; or > 10 years), but they were not asked about age. The physicians were also asked if they needed (*yes/no*) updating and education on NSAIDS.

#### Purchase of NSAIDS

Purchase statistics of NSAIDS in the clinic in 2015 and 2016 (the 2 previous years before the survey) were provided through the hospital pharmacies drug statistics (SLS) in Norway, with specific data from Haukeland Hospital Pharmacy [10]. The SLS contains a complete overview of all drugs purchased by Norwegian hospital units from 2006 to the current date. NSAIDS included in the study were based on to the Anatomical Therapeutic Chemical (ATC) classification system [11], and included ATC-code M01A with the exception of M01A X05 (glucosamin).

#### Statistics and ethics

The survey was conducted by the use of Corporater Surveyor (Helse Vest IKT, Bergen, Norway). SPSS^®^ Statistics for Windows, Version 24.0. Armonk, NY, USA; IBM Corp was used for descriptive data analysis. Participation in the survey was anonymous and voluntary and approved by the head of the clinic.

### Results

#### Participation

Forty-five (55%) of 82 physicians in the orthopedic clinic participated in the survey.

#### Rating

**Fig. 1:**
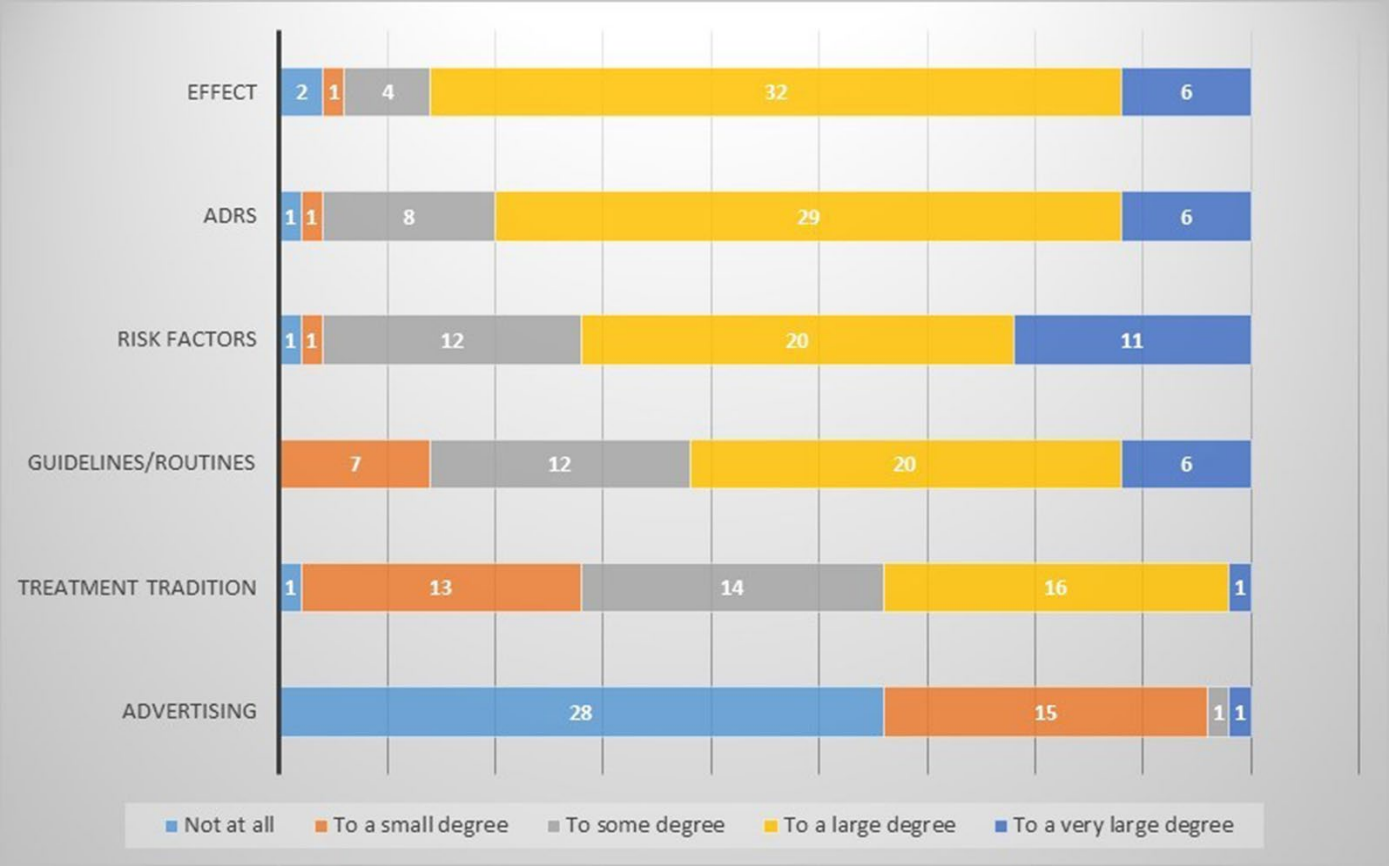
Rating the importance of factors for prescribing of nonsteroidal anti-inflammatory drugs (NSAIDS) among orthopedic physicians (n = 45)

The participants rated effect and ADRS as the most important factors for prescribing, with a low influence from advertising (Fig. 1).

**Fig. 2:**
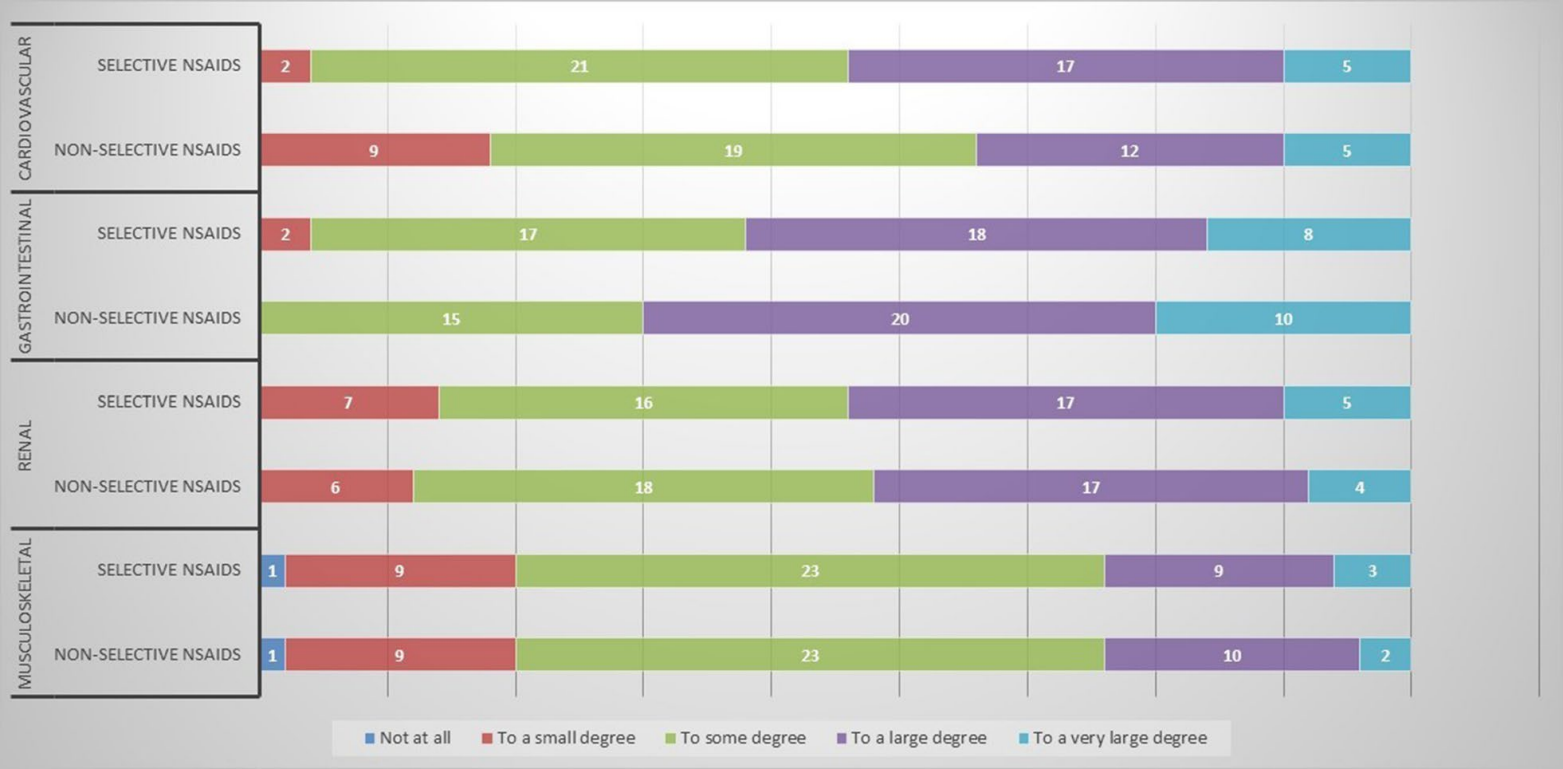
Rating the importance of adverse drug reactions (ADRS) for prescribing of selective or non-selective nonsteroidal anti-inflammatory drugs (NSAIDS) among orthopedic physicians (n = 45). Notice that selective NSAIDS included diclofenac

Participants were equally concerned about ADRS following treatment with non-selective and selective NSAIDS irrespective of type of ADR. They were generally more concerned about cardiovascular, gastrointestinal and renal ADRS than musculoskeletal ADRS (Fig. 2). There was more concern with regard to tissue repair of fractures than soft tissue (Additional file 1: Figure S1).

#### Purchase of NSAIDS

**Fig. 3:**
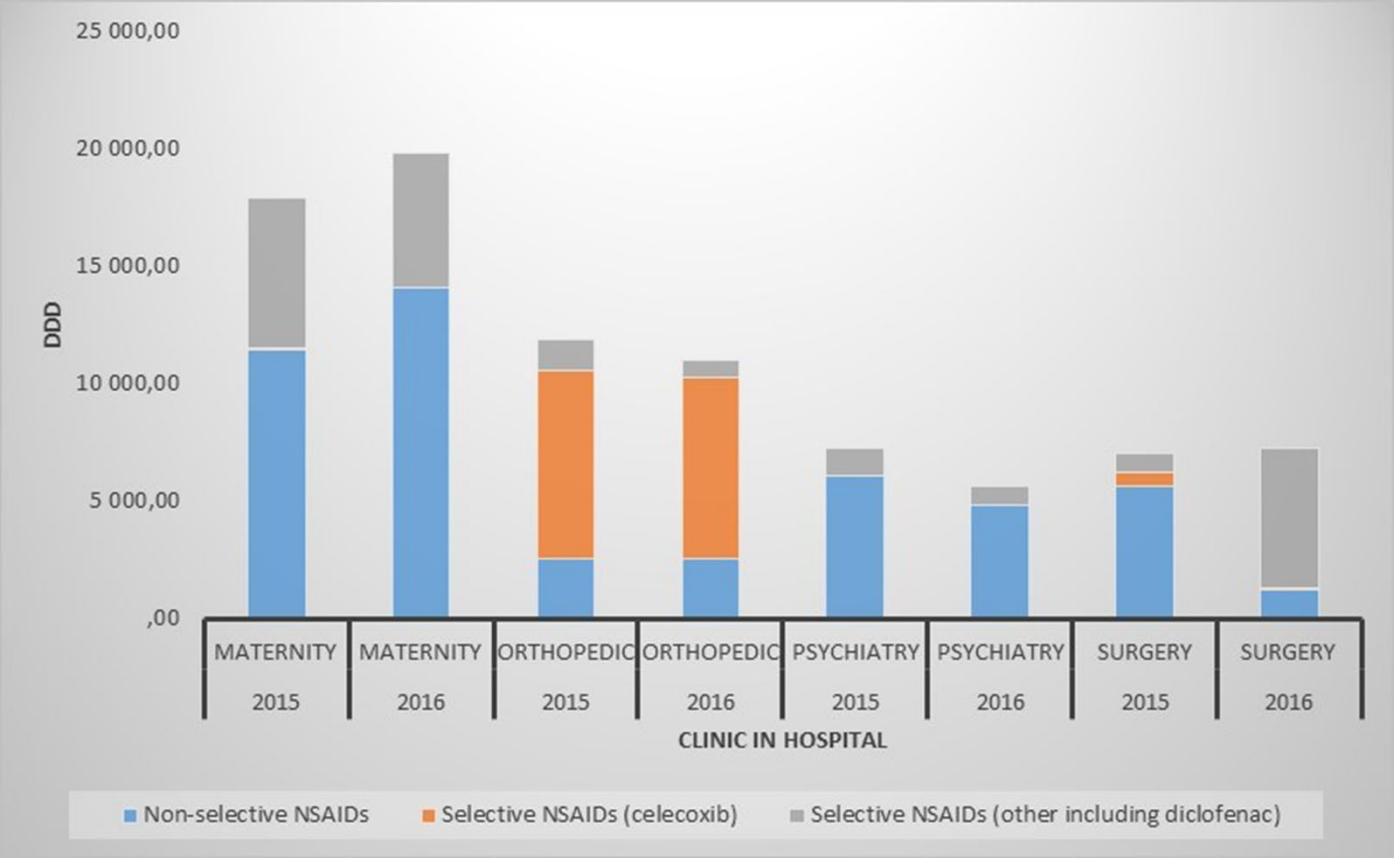
Purchase statistics from 2015 and 2016 in the clinics that used most nonsteroidal anti-inflammatory drugs (NSAIDS) in the university hospital where the survey took place. DDD = Defined Daily Dose: the assumed average maintenance dose per day for a drug used for its main indication in adults

Purchase statistics (Fig. 3) showed that a selective NSAID (celecoxib) dominated in the orthopedic wards, and this was in contrast to other clinics that were top users of NSAIDS in the university hospital.

#### Need of updating and education

Forty-one (91%) of the 45 physicians stated that they needed updating and education on NSAIDS.

### Discussion

This small pilot survey showed a discrepancy between perception of factors important for prescribing, and purchase statistics, of NSAIDS among physicians in an orthopedic clinic. If purchase statistics is used as a proxy for prescribing, the physicians showed a conformity to use of celecoxib. This is in contrast to their stated perception, where effect, ADRS and risk factors were rated as important for prescribing. Furthermore, the participants were equally concerned about non-selective and selective NSAIDS with regard to ADRS although the literature suggest different ADR profiles for the respective classes. A hypothesis of conformity to internal guidelines/routines was also supported when the orthopedic clinic was compared to other clinics in the university hospital with regard to purchase statistics of NSAIDS.

According to purchase statistics in 2015 and 2016, the Orthopedic Clinic in Haukeland University Hospital mainly used celecoxib in contrast to other clinics in the hospital, and also compared to the general use of prescription NSAIDS in Norway [2]. In the period from 2006 to 2011, celecoxib constituted only between 1 and 8% of NSAIDS purchased to the clinic but from 2012 to 2016, celecoxib constituted between 44 and 55%. A possible explanation for this change was a new local routine in the clinic for perioperative pain management in 2012. This routine for multimodal pain management in hip and knee replacement surgery recommends use of paracetamol, celecoxib and gabapentin. The routine was based on guidelines from The American Pain Society and The American Society of Anesthesiologist [3]. Before 2012, epidural anesthesia was used, but due to unpredictable pain management and several cases of nausea and hypotension, it was discarded. Thus, compliance with local routines among the physicians in the orthopedic clinic could be a motivation for the change in purchase statistics in 2012.

In comparison, pain management of patients with hip fracture in another major Norwegian orthopedic clinic included paracetamol regularly and opioids as needed rather than NSAIDS [12]. Data from this prescribing study was collected retrospectively from patient records between 2008 and 2010. Only drug use at admission and at discharge was recorded. Based on recent communication with one of the authors, the local routine in this clinic now recommends NSAIDs as a central choice for analgesia, but purchase statistics from the hospital pharmacy show that paracetamol and opioids are far more often used than NSAIDs [13].

A qualitative study among general practitioners (GPs) in New Zealand found that NSAID prescribing is a complex balance between pragmatism and risk assessments of potential ADRS [14]. GPs were aware of the general risks of NSAIDS but weighed these up against specific risk factors and potential benefits for individual patients. They were most concerned about long-term use, risks for children, older people, and patients with comorbidities. GPs considered gastric, cardiac, and renal risks of patients as well as drug interactions. Mitigation strategies included alternative treatment, choice and dose of NSAID [14].

Internet surveys among US primary care providers (PCPs) compared results from 2003 to 2006 with regard to perceptions and practices with NSAIDS [15]. Fifty-nine per cent of PCPs reported that they prescribed COX‐2 selective NSAIDS less frequently in 2006 compared to 2003. In addition, COX‐2 selective NSAIDS which accounted for over 40% of NSAID recommendations in 2003 accounted for only 25% in 2006. However, over 50% of PCPs did not perceive that celecoxib was associated with increased risk of myocardial infarction [15].

Taken together these studies suggest that the complexity and controversy associated with prescribing of NSAIDS is prevalent in hospitals and outpatient care worldwide. One speculation to explain the results from 2006 in the study among US primary care providers was the low fraction of participants above 65 years (1%), and that that younger physicians were more likely to be aware of recently published medical literature [15]. Notably, 11 participants in our survey had working experience less than five years. However, these participants did not rate risk of ADRS differently than more experienced orthopedics, except that they were more concerned about gastrointestinal ADRS.

Awareness of physician’s attitudes towards pharmaceutical industry is important as it can influence their clinical decision making leading to greater prescriptions of branded drugs over low-cost medicines [16]. A study among GPs in Scotland found adherence to local guidelines to be more important for prescribing of NSAIDS than advertisements [17]. Our results also suggest a weak influence from the pharmaceutical industry but a high conformity to the clinic guidelines.

Physicians in our survey did not separate non-selective and selective NSAIDS with regard to type of ADR. This could reflect the incomplete evidence and controversy in the literature [6–9]. However, their conformity to clinic guidelines with preferred prescribing of celecoxib is in contrast to the advice to find the optimal NSAID for each patient [9, 14]. In this respect, the interest in updating and education on NSAIDS among the participating physicians is promising. Based on our preliminary observations, we suggest that perception of prescribing factors of NSAIDS among orthopedics should be surveyed in multicenter or multinational studies.

## Limitations

The study was limited to a single clinic, with a response rate of 55%. A response rate of 40% was considered reasonable to identify trends in prescribing decisions and to identify potential educational issues in a larger survey that used postal questionnaires [15]. A speculation would be that purchase statistics indicates that non-participants in our study showed the same conformity to clinic guidelines as the participants. Although the survey did not directly measure physicians knowledge of the current literature on NSAIDS, prescribing preferences suggest that there was a potential for drug information efforts in the orthopedic clinic. The high fraction of participants interested in drug information about NSAIDS suggests that several non-participants could perhaps share this motivation.

## Supplementary information

**Additional file 1: Figure S1.** Rating the importance of musculoskeletal ADRS for prescribing of selective or non-selective nonsteroidal anti-inflammatory drugs (NSAIDS) among orthopedic physicians (n = 45). Notice that selective NSAIDS included diclofenac.

## Data Availability

The datasets used in the current study is based on results in Sofie Opskar`s master thesis in pharmacy (in Norwegian) submitted May 2017 to the University of Bergen. Additional data are available from this author on reasonable request. Availability of statistics from hospital pharmacies drug statistics in Norway requires an application to the Norwegian hospital pharmacies (https://sykehusapotekene.no/).

## Abbreviations

ADRS: Adverse drug reactions
COX: Cyclooxygenase
NSAIDS: Nonsteroidal anti-inflammatory drugs
PCPs: US primary care providers
SPSS: Statistical package for the social science
