# Supplementary figures and images for: Perception of prescribing factors and purchase statistics of non-steroidal anti-inflammatory drugs in an orthopedic clinic
Source: BMC Res Notes. 2020 Feb 24;13:100. doi: 10.1186/s13104-020-04949-y (PMC7041082; doi:10.1186/s13104-020-04949-y)

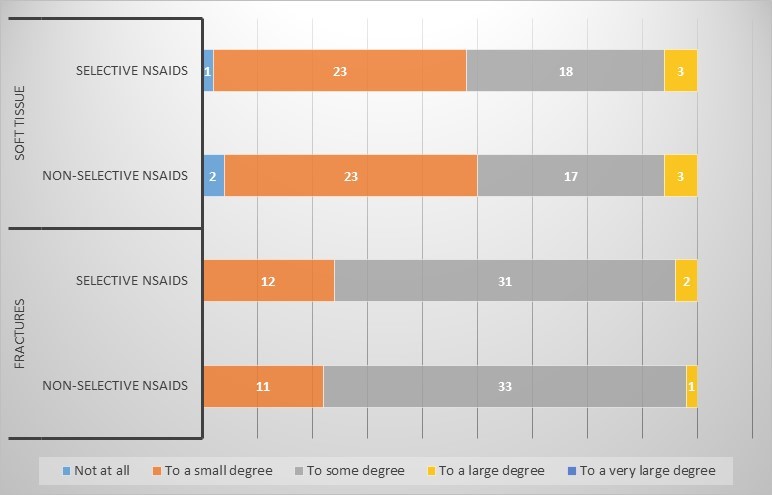

Supplement: Supplementary file 1 — Additional file 1: Figure S1. Rating the importance of musculoskeletal ADRS for prescribing of selective or non-selective nonsteroidal anti-inflammatory drugs (NSAIDS) among orthopedic physicians (n = 45). Notice that selective NSAIDS included diclofenac. [file 13104_2020_4949_MOESM1_ESM.jpg]
